# Supplementary material for: Single Cell Transcriptomic and Chromatin Profiles Suggest Layer Vb Is the Only Layer With Shared Excitatory Cell Types in the Medial and Lateral Entorhinal Cortex
Source: Front Neural Circuits. 2022 Jan 26;15:806154. doi: 10.3389/fncir.2021.806154 (PMC8826650; doi:10.3389/fncir.2021.806154)
Supplement: Supplementary file 14 [file Data_Sheet_1.ZIP › 8/knownResults.html]

8 - Homer Known Motif Enrichment Results


# Homer Known Motif Enrichment Results (8)

Homer *de novo* Motif Results  
Gene Ontology Enrichment Results  
Known Motif Enrichment Results (txt file)  
Total Target Sequences = 18997, Total Background Sequences = 30521

|  |  |  |  |  |  |  |  |  |  |  |  |
| --- | --- | --- | --- | --- | --- | --- | --- | --- | --- | --- | --- |
| Rank | Motif | Name | P-value | log P-pvalue | q-value (Benjamini) | # Target Sequences with Motif | % of Targets Sequences with Motif | # Background Sequences with Motif | % of Background Sequences with Motif | Motif File | SVG |
| 1 | A T G C T G C A A G T C C G T A A G T C A G T C A C G T A C T G A T G C G T C A | E2A(bHLH),near\_PU.1/Bcell-PU.1-ChIP-Seq(GSE21512)/Homer | 1e-2217 | -5.106e+03 | 0.0000 | 9977.0 | 52.52% | 5969.9 | 19.56% | motif file (matrix) | svg |
| 2 | T G A C A T G C C G T A A T C G A T G C C A G T C A T G A C T G A G T C G T A C | HEB(bHLH)/mES-Heb-ChIP-Seq(GSE53233)/Homer | 1e-2157 | -4.968e+03 | 0.0000 | 11279.0 | 59.37% | 7685.2 | 25.18% | motif file (matrix) | svg |
| 3 | C G T A T G A C T C G A A G T C C G T A A T C G A T G C A C G T A C T G A G T C | E2A(bHLH)/proBcell-E2A-ChIP-Seq(GSE21978)/Homer | 1e-2034 | -4.685e+03 | 0.0000 | 9601.0 | 50.54% | 5861.4 | 19.20% | motif file (matrix) | svg |
| 4 | T C G A A G T C C G T A A T C G T A G C A C G T A C T G A G C T A C G T A G T C | Ptf1a(bHLH)/Panc1-Ptf1a-ChIP-Seq(GSE47459)/Homer | 1e-1872 | -4.312e+03 | 0.0000 | 13453.0 | 70.82% | 11473.6 | 37.59% | motif file (matrix) | svg |
| 5 | A G C T T C G A G T A C T C G A A T G C A T G C G C A T A T C G A G T C A G C T | Snail1(Zf)/LS174T-SNAIL1.HA-ChIP-Seq(GSE127183)/Homer | 1e-1839 | -4.236e+03 | 0.0000 | 7121.0 | 37.48% | 3545.4 | 11.62% | motif file (matrix) | svg |
| 6 | T G C A G T A C C G T A A T C G A C T G A C G T C T A G C G A T T C G A A G T C | ZEB1(Zf)/PDAC-ZEB1-ChIP-Seq(GSE64557)/Homer | 1e-1573 | -3.623e+03 | 0.0000 | 10114.0 | 53.24% | 7454.0 | 24.42% | motif file (matrix) | svg |
| 7 | T A C G C T A G T A C G A G T C C G T A A G T C A G T C A C G T A C T G A G T C G A T C T A G C | Slug(Zf)/Mesoderm-Snai2-ChIP-Seq(GSE61475)/Homer | 1e-1489 | -3.429e+03 | 0.0000 | 5152.0 | 27.12% | 2232.3 | 7.31% | motif file (matrix) | svg |
| 8 | T A C G T A C G C T A G T C A G A G T C C G T A A T C G A T G C A C G T A C T G A G T C G A C T | Ascl2(bHLH)/ESC-Ascl2-ChIP-Seq(GSE97712)/Homer | 1e-1483 | -3.415e+03 | 0.0000 | 7491.0 | 39.43% | 4521.9 | 14.81% | motif file (matrix) | svg |
| 9 | C T A G A G T C T A C G T A C G T G A C C G T A A C T G T A G C G C A T C A T G A T G C A G C T | Ascl1(bHLH)/NeuralTubes-Ascl1-ChIP-Seq(GSE55840)/Homer | 1e-1314 | -3.028e+03 | 0.0000 | 8728.0 | 45.94% | 6319.0 | 20.70% | motif file (matrix) | svg |
| 10 | C T A G A C T G T G C A A G T C C G T A A C T G A C T G A C G T C T A G C G A T T A C G A G T C | ZEB2(Zf)/SNU398-ZEB2-ChIP-Seq(GSE103048)/Homer | 1e-1076 | -2.480e+03 | 0.0000 | 6054.0 | 31.87% | 3773.6 | 12.36% | motif file (matrix) | svg |
| 11 | T C G A T G A C G T A C C G T A C A G T T G A C A C G T A C T G A G C T A G C T | NeuroG2(bHLH)/Fibroblast-NeuroG2-ChIP-Seq(GSE75910)/Homer | 1e-721 | -1.662e+03 | 0.0000 | 8050.0 | 42.38% | 7153.5 | 23.44% | motif file (matrix) | svg |
| 12 | T C A G A C G T T C G A T A G C A G T C C G T A A C T G G T A C A C G T A C T G A T C G A G T C | Atoh1(bHLH)/Cerebellum-Atoh1-ChIP-Seq(GSE22111)/Homer | 1e-697 | -1.605e+03 | 0.0000 | 6005.0 | 31.61% | 4643.0 | 15.21% | motif file (matrix) | svg |
| 13 | T G A C A G T C C G T A A C T G G T A C A C G T A C T G A C G T G A C T G A T C | Twist2(bHLH)/Myoblast-Twist2.Ty1-ChIP-Seq(GSE127998)/Homer | 1e-661 | -1.524e+03 | 0.0000 | 8801.0 | 46.33% | 8403.2 | 27.53% | motif file (matrix) | svg |
| 14 | T C A G T G A C G T A C C G T A A C G T T G A C A C G T T C A G A G C T G A C T | NeuroD1(bHLH)/Islet-NeuroD1-ChIP-Seq(GSE30298)/Homer | 1e-636 | -1.466e+03 | 0.0000 | 4930.0 | 25.95% | 3560.3 | 11.66% | motif file (matrix) | svg |
| 15 | C T A G T C G A T G A C A G T C C G T A A C T G G T A C A C G T A C T G A C T G | BHLHA15(bHLH)/NIH3T3-BHLHB8.HA-ChIP-Seq(GSE119782)/Homer | 1e-631 | -1.455e+03 | 0.0000 | 7305.0 | 38.45% | 6487.3 | 21.25% | motif file (matrix) | svg |
| 16 | C T G A T G A C T G A C C G T A A C G T T G A C A G C T C T A G A C G T G A C T | Olig2(bHLH)/Neuron-Olig2-ChIP-Seq(GSE30882)/Homer | 1e-615 | -1.418e+03 | 0.0000 | 9372.0 | 49.33% | 9403.1 | 30.81% | motif file (matrix) | svg |
| 17 | T A C G T G C A A G T C C G T A A C G T T G A C A C G T A C T G A C T G G C A T | TCF4(bHLH)/SHSY5Y-TCF4-ChIP-Seq(GSE96915)/Homer | 1e-560 | -1.291e+03 | 0.0000 | 7769.0 | 40.90% | 7381.0 | 24.18% | motif file (matrix) | svg |
| 18 | T C G A A G T C C G T A A T C G A T G C C G A T A C T G A G T C A G C T A C T G | Tcf12(bHLH)/GM12878-Tcf12-ChIP-Seq(GSE32465)/Homer | 1e-489 | -1.126e+03 | 0.0000 | 4668.0 | 24.57% | 3683.8 | 12.07% | motif file (matrix) | svg |
| 19 | T C G A T C G A A G T C C G T A C T A G T A G C A C G T A C T G | MyoG(bHLH)/C2C12-MyoG-ChIP-Seq(GSE36024)/Homer | 1e-438 | -1.011e+03 | 0.0000 | 4968.0 | 26.15% | 4212.5 | 13.80% | motif file (matrix) | svg |
| 20 | C T G A T C A G G T A C G C T A A C T G T G A C G C A T C A T G | SCL(bHLH)/HPC7-Scl-ChIP-Seq(GSE13511)/Homer | 1e-401 | -9.255e+02 | 0.0000 | 14289.0 | 75.22% | 18455.2 | 60.46% | motif file (matrix) | svg |
| 21 | A C G T T C G A T C G A A G T C G T C A T A C G A T G C A C G T A C T G A G C T | Myf5(bHLH)/GM-Myf5-ChIP-Seq(GSE24852)/Homer | 1e-323 | -7.453e+02 | 0.0000 | 3524.0 | 18.55% | 2880.1 | 9.44% | motif file (matrix) | svg |
| 22 | C G A T T G C A T G C A G A T C C G T A A C T G T G A C G A C T C A T G A C T G | Tcf21(bHLH)/ArterySmoothMuscle-Tcf21-ChIP-Seq(GSE61369)/Homer | 1e-306 | -7.059e+02 | 0.0000 | 4275.0 | 22.50% | 3859.5 | 12.64% | motif file (matrix) | svg |
| 23 | C T G A C T A G A T C G A G C T A C T G G A C T A G T C C T G A | Tbx5(T-box)/HL1-Tbx5.biotin-ChIP-Seq(GSE21529)/Homer | 1e-236 | -5.448e+02 | 0.0000 | 10693.0 | 56.29% | 13549.0 | 44.39% | motif file (matrix) | svg |
| 24 | C A T G A G T C G A C T C G T A C G A T G C A T G A C T G C A T C G A T C T A G C A T G T G A C | Mef2b(MADS)/HEK293-Mef2b.V5-ChIP-Seq(GSE67450)/Homer | 1e-226 | -5.215e+02 | 0.0000 | 4134.0 | 21.76% | 4042.7 | 13.24% | motif file (matrix) | svg |
| 25 | G T C A T G C A G C T A A G T C C G T A A C T G T G A C G C A T T C A G C A G T | Ap4(bHLH)/AML-Tfap4-ChIP-Seq(GSE45738)/Homer | 1e-206 | -4.764e+02 | 0.0000 | 4924.0 | 25.92% | 5201.9 | 17.04% | motif file (matrix) | svg |
| 26 | A G C T A C G T A C T G A T G C A G T C C G T A C T G A T A C G | NF1-halfsite(CTF)/LNCaP-NF1-ChIP-Seq(Unpublished)/Homer | 1e-168 | -3.869e+02 | 0.0000 | 6098.0 | 32.10% | 7109.3 | 23.29% | motif file (matrix) | svg |
| 27 | C T G A T C A G A G T C C G T A A T C G A T G C C G A T A C T G A G T C G A C T A T C G A G T C | MyoD(bHLH)/Myotube-MyoD-ChIP-Seq(GSE21614)/Homer | 1e-155 | -3.575e+02 | 0.0000 | 3015.0 | 15.87% | 2960.1 | 9.70% | motif file (matrix) | svg |
| 28 | C A T G C T A G T C G A A C G T A C T G C G T A T A G C C G A T T G A C C G T A A G C T G A T C | Fra2(bZIP)/Striatum-Fra2-ChIP-Seq(GSE43429)/Homer | 1e-146 | -3.373e+02 | 0.0000 | 2072.0 | 10.91% | 1828.0 | 5.99% | motif file (matrix) | svg |
| 29 | A C T G C T A G T C G A C G A T C A T G G C T A A T C G C G A T G T A C G C T A A G C T G T A C | Fra1(bZIP)/BT549-Fra1-ChIP-Seq(GSE46166)/Homer | 1e-145 | -3.357e+02 | 0.0000 | 2381.0 | 12.53% | 2207.0 | 7.23% | motif file (matrix) | svg |
| 30 | C A T G G T A C G A C T G C T A C G T A C G T A C G T A G C T A G A C T C T G A T C A G G T A C | Mef2c(MADS)/GM12878-Mef2c-ChIP-Seq(GSE32465)/Homer | 1e-145 | -3.357e+02 | 0.0000 | 2439.0 | 12.84% | 2279.3 | 7.47% | motif file (matrix) | svg |
| 31 | C T A G T C G A C G A T A C T G C G T A T A C G A G C T T G A C G C T A A C G T G A T C T A G C | Fosl2(bZIP)/3T3L1-Fosl2-ChIP-Seq(GSE56872)/Homer | 1e-143 | -3.304e+02 | 0.0000 | 1497.0 | 7.88% | 1170.4 | 3.83% | motif file (matrix) | svg |
| 32 | C T A G T C G A A C G T A C T G C G T A A T G C A C G T G T A C C G T A A G C T G A T C G T A C | Atf3(bZIP)/GBM-ATF3-ChIP-Seq(GSE33912)/Homer | 1e-141 | -3.247e+02 | 0.0000 | 2713.0 | 14.28% | 2648.8 | 8.68% | motif file (matrix) | svg |
| 33 | G T A C G A C T C G T A C T G A T C G A C G T A G C T A C A G T C T G A T A C G | Mef2a(MADS)/HL1-Mef2a.biotin-ChIP-Seq(GSE21529)/Homer | 1e-135 | -3.114e+02 | 0.0000 | 2100.0 | 11.05% | 1910.5 | 6.26% | motif file (matrix) | svg |
| 34 | A C T G C A T G T C G A A C G T A C T G C G T A A T C G A C G T G T A C C G T A G A C T A G T C | Fos(bZIP)/TSC-Fos-ChIP-Seq(GSE110950)/Homer | 1e-133 | -3.078e+02 | 0.0000 | 2480.0 | 13.05% | 2390.7 | 7.83% | motif file (matrix) | svg |
| 35 | T C G A A C G T A C G T C T G A G A T C T C A G G A C T G T C A C G T A A G C T G T C A C T A G A G C T A C G T T C G A | NFIL3(bZIP)/HepG2-NFIL3-ChIP-Seq(Encode)/Homer | 1e-131 | -3.021e+02 | 0.0000 | 2732.0 | 14.38% | 2725.4 | 8.93% | motif file (matrix) | svg |
| 36 | C T A G T C G A G C A T C A T G G C T A T A G C C G A T G T A C C T G A A G C T | JunB(bZIP)/DendriticCells-Junb-ChIP-Seq(GSE36099)/Homer | 1e-129 | -2.986e+02 | 0.0000 | 2277.0 | 11.99% | 2155.8 | 7.06% | motif file (matrix) | svg |
| 37 | C A G T T G C A A C G T A C T G C G T A A T C G C G A T T G A C C G T A A C G T | BATF(bZIP)/Th17-BATF-ChIP-Seq(GSE39756)/Homer | 1e-126 | -2.913e+02 | 0.0000 | 2701.0 | 14.22% | 2711.2 | 8.88% | motif file (matrix) | svg |
| 38 | C T A G T C G A A C G T A C T G C G T A T A G C C G A T G T A C C G T A A G C T G A T C G T A C | Jun-AP1(bZIP)/K562-cJun-ChIP-Seq(GSE31477)/Homer | 1e-121 | -2.796e+02 | 0.0000 | 1149.0 | 6.05% | 858.1 | 2.81% | motif file (matrix) | svg |
| 39 | T C G A A C G T C A T G G C T A T A G C C G A T G T A C G C T A A C G T A T G C | AP-1(bZIP)/ThioMac-PU.1-ChIP-Seq(GSE21512)/Homer | 1e-121 | -2.791e+02 | 0.0000 | 2917.0 | 15.36% | 3024.4 | 9.91% | motif file (matrix) | svg |
| 40 | C T A G A G C T G A C T C A T G A G T C A G T C G T C A C A G T C T A G T C A G G T A C C T G A T C G A G A T C T G A C | Rfx2(HTH)/LoVo-RFX2-ChIP-Seq(GSE49402)/Homer | 1e-100 | -2.319e+02 | 0.0000 | 551.0 | 2.90% | 306.4 | 1.00% | motif file (matrix) | svg |
| 41 | T G A C G T A C C G T A A C T G T G A C C G A T A C T G A T C G A G C T T A C G T C G A T A G C G T A C C G T A A T C G T G A C G C A T A C T G A C T G A T G C | Twist(bHLH)/HMLE-TWIST1-ChIP-Seq(Chang\_et\_al)/Homer | 1e-94 | -2.186e+02 | 0.0000 | 785.0 | 4.13% | 549.2 | 1.80% | motif file (matrix) | svg |
| 42 | T C A G G A C T C A G T C T G A A G C T C T A G G A C T T G C A C T G A A G T C | HLF(bZIP)/HSC-HLF.Flag-ChIP-Seq(GSE69817)/Homer | 1e-92 | -2.134e+02 | 0.0000 | 3491.0 | 18.38% | 4002.0 | 13.11% | motif file (matrix) | svg |
| 43 | C A T G A G T C A G C T C G T A C G A T C G A T G C A T G C A T C G A T C T G A C A T G T G A C | Mef2d(MADS)/Retina-Mef2d-ChIP-Seq(GSE61391)/Homer | 1e-89 | -2.063e+02 | 0.0000 | 1029.0 | 5.42% | 832.3 | 2.73% | motif file (matrix) | svg |
| 44 | G A C T C T A G A T G C A G T C G T C A T A C G A T G C A T C G | HIC1(Zf)/Treg-ZBTB29-ChIP-Seq(GSE99889)/Homer | 1e-88 | -2.033e+02 | 0.0000 | 6340.0 | 33.37% | 8182.7 | 26.81% | motif file (matrix) | svg |
| 45 | A G T C A T C G C T A G A G C T G A C T C T A G A G T C A G T C G C T A C A G T T C A G T C A G G A T C C T G A T C G A G A T C | RFX(HTH)/K562-RFX3-ChIP-Seq(SRA012198)/Homer | 1e-86 | -1.986e+02 | 0.0000 | 486.0 | 2.56% | 275.9 | 0.90% | motif file (matrix) | svg |
| 46 | A G C T C A T G G C A T G A T C T G C A C T A G G A T C A C G T | Tgif2(Homeobox)/mES-Tgif2-ChIP-Seq(GSE55404)/Homer | 1e-83 | -1.912e+02 | 0.0000 | 10325.0 | 54.35% | 14447.9 | 47.33% | motif file (matrix) | svg |
| 47 | C A T G C T A G A G C T G A C T C A T G A G T C G A T C G C T A C G A T C T A G T C A G G T A C C T G A T C G A | X-box(HTH)/NPC-H3K4me1-ChIP-Seq(GSE16256)/Homer | 1e-68 | -1.576e+02 | 0.0000 | 613.0 | 3.23% | 445.6 | 1.46% | motif file (matrix) | svg |
| 48 | T C G A A C T G A C T G C G T A C G T A T C G A A G T C C T G A A T C G G T A C G C A T C A T G | ETS:E-box(ETS,bHLH)/HPC7-Scl-ChIP-Seq(GSE22178)/Homer | 1e-61 | -1.425e+02 | 0.0000 | 470.0 | 2.47% | 315.0 | 1.03% | motif file (matrix) | svg |
| 49 | C T A G C T G A T C A G A T C G A G C T C A T G G A C T A G T C C T G A T G C A | Tbx6(T-box)/ESC-Tbx6-ChIP-Seq(GSE93524)/Homer | 1e-59 | -1.360e+02 | 0.0000 | 3563.0 | 18.76% | 4410.5 | 14.45% | motif file (matrix) | svg |
| 50 | A C T G A G T C G T C A C G T A A G T C C G T A C T A G C T A G G A C T C A T G | SCRT1(Zf)/HEK293-SCRT1.eGFP-ChIP-Seq(Encode)/Homer | 1e-58 | -1.352e+02 | 0.0000 | 1168.0 | 6.15% | 1135.5 | 3.72% | motif file (matrix) | svg |
| 51 | G A C T C G A T C T G A G T C A G A C T C G A T T C G A C G T A G C T A G C T A T G A C G T A C C G T A A C T G T G C A C G A T A C T G A C G T | Pitx1:Ebox(Homeobox,bHLH)/Hindlimb-Pitx1-ChIP-Seq(GSE41591)/Homer | 1e-57 | -1.324e+02 | 0.0000 | 1012.0 | 5.33% | 948.0 | 3.11% | motif file (matrix) | svg |
| 52 | A C T G T C A G A G C T G A C T C A T G A G T C A G T C G C T A C G A T C T A G T C A G G T A C C T G A T C G A | Rfx1(HTH)/NPC-H3K4me1-ChIP-Seq(GSE16256)/Homer | 1e-52 | -1.220e+02 | 0.0000 | 859.0 | 4.52% | 784.8 | 2.57% | motif file (matrix) | svg |
| 53 | C A G T T C A G A G C T G A C T A C G T A G T C G A T C G A C T C T G A A C T G G A T C C G T A C T G A A G T C G T A C | Rfx6(HTH)/Min6b1-Rfx6.HA-ChIP-Seq(GSE62844)/Homer | 1e-50 | -1.163e+02 | 0.0000 | 3549.0 | 18.68% | 4484.8 | 14.69% | motif file (matrix) | svg |
| 54 | C A T G A C T G C T A G T C G A T C G A T C G A T C G A T C A G T C A G T C A G T G A C T G A C C G T A A C T G T G C A C G A T A C T G | RBPJ:Ebox(?,bHLH)/Panc1-Rbpj1-ChIP-Seq(GSE47459)/Homer | 1e-48 | -1.123e+02 | 0.0000 | 1309.0 | 6.89% | 1375.5 | 4.51% | motif file (matrix) | svg |
| 55 | T A C G A T C G T A G C G A T C A C T G A C G T A G T C A C G T C T A G A T C G | Smad4(MAD)/ESC-SMAD4-ChIP-Seq(GSE29422)/Homer | 1e-48 | -1.106e+02 | 0.0000 | 5083.0 | 26.76% | 6789.8 | 22.24% | motif file (matrix) | svg |
| 56 | T A G C G T A C C G T A C T A G A C T G T G C A C G T A A T G C C G T A A T C G | AR-halfsite(NR)/LNCaP-AR-ChIP-Seq(GSE27824)/Homer | 1e-42 | -9.742e+01 | 0.0000 | 11482.0 | 60.44% | 16947.1 | 55.52% | motif file (matrix) | svg |
| 57 | C G A T T A C G T G A C G A C T C A T G C G T A T A C G A C G T G T A C C T G A | Bach2(bZIP)/OCILy7-Bach2-ChIP-Seq(GSE44420)/Homer | 1e-41 | -9.503e+01 | 0.0000 | 776.0 | 4.08% | 741.2 | 2.43% | motif file (matrix) | svg |
| 58 | G A C T G C A T C T A G C G A T G A T C T C G A C A T G G A T C | Tgif1(Homeobox)/mES-Tgif1-ChIP-Seq(GSE55404)/Homer | 1e-37 | -8.740e+01 | 0.0000 | 9471.0 | 49.86% | 13787.0 | 45.17% | motif file (matrix) | svg |
| 59 | G T A C G A T C C A G T A G T C A G T C A G T C T G C A G A T C C T G A A T G C G T C A A C G T | WT1(Zf)/Kidney-WT1-ChIP-Seq(GSE90016)/Homer | 1e-37 | -8.626e+01 | 0.0000 | 1435.0 | 7.55% | 1627.8 | 5.33% | motif file (matrix) | svg |
| 60 | G C T A G A C T G A C T T G C A C G T A A G T C C G T A T A G C G A T C G A C T | Eomes(T-box)/H9-Eomes-ChIP-Seq(GSE26097)/Homer | 1e-35 | -8.236e+01 | 0.0000 | 6893.0 | 36.28% | 9763.8 | 31.99% | motif file (matrix) | svg |
| 61 | C G T A A C G T A G C T C G A T C T A G G T A C C G T A A G C T C G T A G C T A | Oct4(POU,Homeobox)/mES-Oct4-ChIP-Seq(GSE11431)/Homer | 1e-34 | -7.883e+01 | 0.0000 | 2078.0 | 10.94% | 2553.0 | 8.36% | motif file (matrix) | svg |
| 62 | G A C T C A G T A G C T C G A T A G T C G A T C A G T C C G T A A T G C T C A G | Rbpj1(?)/Panc1-Rbpj1-ChIP-Seq(GSE47459)/Homer | 1e-34 | -7.880e+01 | 0.0000 | 4722.0 | 24.86% | 6454.5 | 21.15% | motif file (matrix) | svg |
| 63 | A G C T G C A T A C T G A C G T A G T C A C G T C T A G T A C G | Smad3(MAD)/NPC-Smad3-ChIP-Seq(GSE36673)/Homer | 1e-33 | -7.751e+01 | 0.0000 | 8776.0 | 46.20% | 12765.7 | 41.82% | motif file (matrix) | svg |
| 64 | G A C T C G T A A C G T A C T G A G T C C G T A C T G A C G T A C A G T A C G T G T C A T C A G | Brn1(POU,Homeobox)/NPC-Brn1-ChIP-Seq(GSE35496)/Homer | 1e-32 | -7.562e+01 | 0.0000 | 1511.0 | 7.95% | 1772.5 | 5.81% | motif file (matrix) | svg |
| 65 | C T G A A G C T A C G T A C G T A G T C G A C T G A C T C T G A C T G A C T A G C G T A C G T A | STAT6(Stat)/CD4-Stat6-ChIP-Seq(GSE22104)/Homer | 1e-30 | -6.937e+01 | 0.0000 | 2009.0 | 10.58% | 2501.3 | 8.19% | motif file (matrix) | svg |
| 66 | G C A T C G T A C G A T A C T G A G T C G C T A C T G A C G T A C A G T A C T G C G T A T C A G | Oct6(POU,Homeobox)/NPC-Pou3f1-ChIP-Seq(GSE35496)/Homer | 1e-29 | -6.883e+01 | 0.0000 | 1904.0 | 10.02% | 2354.4 | 7.71% | motif file (matrix) | svg |
| 67 | C T G A C T A G A C T G G C A T C T A G G C A T A T C G C T G A C G T A G T C A | Tbx21(T-box)/GM12878-TBX21-ChIP-Seq(Encode)/Homer | 1e-29 | -6.823e+01 | 0.0000 | 3297.0 | 17.36% | 4388.5 | 14.38% | motif file (matrix) | svg |
| 68 | C A G T T C A G G A T C A C T G A C G T C T A G A C T G A C T G G A C T C T A G | Egr1(Zf)/K562-Egr1-ChIP-Seq(GSE32465)/Homer | 1e-29 | -6.720e+01 | 0.0000 | 1575.0 | 8.29% | 1897.1 | 6.21% | motif file (matrix) | svg |
| 69 | A T G C G A C T A C T G C A G T G A T C A C G T T A C G T A C G | Smad2(MAD)/ES-SMAD2-ChIP-Seq(GSE29422)/Homer | 1e-28 | -6.644e+01 | 0.0000 | 4810.0 | 25.32% | 6681.7 | 21.89% | motif file (matrix) | svg |
| 70 | A G T C G A C T C A G T A C T G C T A G T G A C G C T A A T G C G C A T A T C G C G A T A C T G G A T C G T A C G T C A C T G A | NF1(CTF)/LNCAP-NF1-ChIP-Seq(Unpublished)/Homer | 1e-28 | -6.608e+01 | 0.0000 | 1068.0 | 5.62% | 1203.0 | 3.94% | motif file (matrix) | svg |
| 71 | G C T A A G C T T A C G G T C A C T G A C G A T C T G A G C A T C A G T A G T C | Brn2(POU,Homeobox)/NPC-Brn2-ChIP-Seq(GSE35496)/Homer | 1e-28 | -6.538e+01 | 0.0000 | 514.0 | 2.71% | 486.8 | 1.59% | motif file (matrix) | svg |
| 72 | G A C T C A G T G A T C G A T C A C G T G A T C C T G A T A C G C G T A G T C A | STAT6(Stat)/Macrophage-Stat6-ChIP-Seq(GSE38377)/Homer | 1e-28 | -6.531e+01 | 0.0000 | 2007.0 | 10.56% | 2519.5 | 8.25% | motif file (matrix) | svg |
| 73 | T G A C G C T A T G A C C G T A T C A G G A T C C G T A C A T G C A T G C T A G C T A G C T A G | Unknown-ESC-element(?)/mES-Nanog-ChIP-Seq(GSE11724)/Homer | 1e-27 | -6.238e+01 | 0.0000 | 1729.0 | 9.10% | 2137.2 | 7.00% | motif file (matrix) | svg |
| 74 | A C T G G T C A A C G T G C A T C G A T T C A G G T A C G T C A A C G T C T G A | Oct11(POU,Homeobox)/NCIH1048-POU2F3-ChIP-seq(GSE115123)/Homer | 1e-26 | -6.049e+01 | 0.0000 | 1514.0 | 7.97% | 1842.8 | 6.04% | motif file (matrix) | svg |
| 75 | A C T G T G A C G T A C C G T A A G T C T A C G A C G T A C T G G T C A A G T C | NPAS2(bHLH)/Liver-NPAS2-ChIP-Seq(GSE39860)/Homer | 1e-25 | -5.799e+01 | 0.0000 | 3542.0 | 18.65% | 4827.0 | 15.81% | motif file (matrix) | svg |
| 76 | T C A G A G C T A C G T A C G T G T A C G A T C C G T A C T A G C A T G G T C A C G T A T C G A | STAT4(Stat)/CD4-Stat4-ChIP-Seq(GSE22104)/Homer | 1e-24 | -5.687e+01 | 0.0000 | 3241.0 | 17.06% | 4383.2 | 14.36% | motif file (matrix) | svg |
| 77 | A T G C T C G A A G T C A G C T A C G T G T A C A G T C G C T A C T A G C A T G G T C A C T G A T C A G A G T C | Stat3+il21(Stat)/CD4-Stat3-ChIP-Seq(GSE19198)/Homer | 1e-23 | -5.462e+01 | 0.0000 | 2266.0 | 11.93% | 2955.3 | 9.68% | motif file (matrix) | svg |
| 78 | C A T G T A C G T A G C G A T C G A T C A T G C G T A C G A C T T C A G A T G C C G A T A T C G C A G T A C T G G T A C | Zic3(Zf)/mES-Zic3-ChIP-Seq(GSE37889)/Homer | 1e-23 | -5.435e+01 | 0.0000 | 1399.0 | 7.36% | 1709.4 | 5.60% | motif file (matrix) | svg |
| 79 | A G T C A C G T A C T G A G C T A C G T A C G T G T C A A G T C | Foxo1(Forkhead)/RAW-Foxo1-ChIP-Seq(Fan\_et\_al.)/Homer | 1e-23 | -5.402e+01 | 0.0000 | 6250.0 | 32.90% | 9013.7 | 29.53% | motif file (matrix) | svg |
| 80 | C G T A C T A G C A T G A G C T A C T G C G A T A T C G C G T A G T C A G T C A | Tbet(T-box)/CD8-Tbet-ChIP-Seq(GSE33802)/Homer | 1e-21 | -4.921e+01 | 0.0000 | 3476.0 | 18.30% | 4795.8 | 15.71% | motif file (matrix) | svg |
| 81 | G C T A A C G T C T A G G T C A C G T A A C G T C G T A C G A T C A G T A G T C C G T A A C G T C T A G C T G A A T C G | OCT:OCT(POU,Homeobox)/NPC-Brn1-ChIP-Seq(GSE35496)/Homer | 1e-21 | -4.860e+01 | 0.0000 | 91.0 | 0.48% | 44.5 | 0.15% | motif file (matrix) | svg |
| 82 | T G C A C T G A A T G C G T C A A C G T A T G C A C G T A C T G A C T G T G C A | ZBTB18(Zf)/HEK293-ZBTB18.GFP-ChIP-Seq(GSE58341)/Homer | 1e-20 | -4.781e+01 | 0.0000 | 1727.0 | 9.09% | 2212.7 | 7.25% | motif file (matrix) | svg |
| 83 | T A G C T C A G C A T G G C A T A G C T C G A T A T G C C G T A C G T A G T C A | CHR(?)/Hela-CellCycle-Expression/Homer | 1e-20 | -4.718e+01 | 0.0000 | 2310.0 | 12.16% | 3068.6 | 10.05% | motif file (matrix) | svg |
| 84 | C T G A C G A T C T A G T C A G G A T C C T G A T C A G G A T C C T G A A C T G A G T C G C T A A C G T A G T C G C A T | PRDM9(Zf)/Testis-DMC1-ChIP-Seq(GSE35498)/Homer | 1e-20 | -4.700e+01 | 0.0000 | 1414.0 | 7.44% | 1767.5 | 5.79% | motif file (matrix) | svg |
| 85 | A G T C G A C T C A G T G T A C A G T C A T C G T C A G A C T G G T C A C G T A | Stat3(Stat)/mES-Stat3-ChIP-Seq(GSE11431)/Homer | 1e-18 | -4.278e+01 | 0.0000 | 1542.0 | 8.12% | 1974.6 | 6.47% | motif file (matrix) | svg |
| 86 | C T A G T C G A C G A T C T A G G C A T C A G T C T A G G A T C C G T A G T C A | CEBP:AP1(bZIP)/ThioMac-CEBPb-ChIP-Seq(GSE21512)/Homer | 1e-16 | -3.831e+01 | 0.0000 | 2472.0 | 13.01% | 3374.3 | 11.05% | motif file (matrix) | svg |
| 87 | T A C G T C G A C A G T A C T G G C T A A T G C C G A T G T A C C G T A A C T G T A G C C G T A | NF-E2(bZIP)/K562-NFE2-ChIP-Seq(GSE31477)/Homer | 1e-16 | -3.732e+01 | 0.0000 | 257.0 | 1.35% | 235.7 | 0.77% | motif file (matrix) | svg |
| 88 | T C G A T A G C G T C A A C T G A C T G C G T A C G T A C T A G A G C T T C A G | ERG(ETS)/VCaP-ERG-ChIP-Seq(GSE14097)/Homer | 1e-15 | -3.678e+01 | 0.0000 | 4667.0 | 24.57% | 6734.3 | 22.06% | motif file (matrix) | svg |
| 89 | T G C A A G C T A C G T C T A G G A T C C T A G G A T C G T C A C T G A A G T C | CEBP(bZIP)/ThioMac-CEBPb-ChIP-Seq(GSE21512)/Homer | 1e-15 | -3.631e+01 | 0.0000 | 2104.0 | 11.08% | 2841.1 | 9.31% | motif file (matrix) | svg |
| 90 | T G C A A G C T C A T G C G T A A G C T A C T G G A T C G T C A C G T A A G C T | Atf4(bZIP)/MEF-Atf4-ChIP-Seq(GSE35681)/Homer | 1e-15 | -3.559e+01 | 0.0000 | 922.0 | 4.85% | 1128.2 | 3.70% | motif file (matrix) | svg |
| 91 | C T G A C T G A C A T G A T C G A G C T A T C G G A C T C A T G C T G A G T C A | Tbr1(T-box)/Cortex-Tbr1-ChIP-Seq(GSE71384)/Homer | 1e-15 | -3.524e+01 | 0.0000 | 4396.0 | 23.14% | 6332.6 | 20.75% | motif file (matrix) | svg |
| 92 | A G T C C G A T A C T G A T C G T G A C G C T A C A T G A T C G T G A C C G A T A C T G T A G C G T A C G T C A | Tlx?(NR)/NPC-H3K4me1-ChIP-Seq(GSE16256)/Homer | 1e-14 | -3.287e+01 | 0.0000 | 1261.0 | 6.64% | 1627.2 | 5.33% | motif file (matrix) | svg |
| 93 | C G T A C G T A C G T A A G T C A G C T C T G A A C T G C T A G A G C T A G T C C G T A T C A G | RORg(NR)/Liver-Rorc-ChIP-Seq(GSE101115)/Homer | 1e-14 | -3.228e+01 | 0.0000 | 324.0 | 1.71% | 330.7 | 1.08% | motif file (matrix) | svg |
| 94 | T G C A T C G A T C G A A C G T C A T G C G T A A G T C T C A G A C G T G T A C C G T A A G C T | CREB5(bZIP)/LNCaP-CREB5.V5-ChIP-Seq(GSE137775)/Homer | 1e-13 | -3.095e+01 | 0.0000 | 1120.0 | 5.90% | 1436.0 | 4.70% | motif file (matrix) | svg |
| 95 | T C A G T A C G T A G C A C G T A C T G C G A T A G T C C G T A T A C G A G T C | Meis1(Homeobox)/MastCells-Meis1-ChIP-Seq(GSE48085)/Homer | 1e-13 | -3.018e+01 | 0.0000 | 5058.0 | 26.63% | 7418.3 | 24.30% | motif file (matrix) | svg |
| 96 | C G T A A C G T A C T G G T A C C G T A A C G T C G T A C G T A A C G T A C T G A G T C C G T A A C G T C T G A G C A T | OCT:OCT-short(POU,Homeobox)/NPC-OCT6-ChIP-Seq(GSE43916)/Homer | 1e-12 | -2.900e+01 | 0.0000 | 2647.0 | 13.93% | 3719.5 | 12.19% | motif file (matrix) | svg |
| 97 | C G T A A C G T A C G T A C G T A C G T A G T C A G T C C T G A A G C T A G C T | NFAT(RHD)/Jurkat-NFATC1-ChIP-Seq(Jolma\_et\_al.)/Homer | 1e-12 | -2.806e+01 | 0.0000 | 3073.0 | 16.18% | 4377.7 | 14.34% | motif file (matrix) | svg |
| 98 | A G T C G T A C A G C T C T A G A G T C C G A T A C T G C G T A A C T G G T C A | Zic(Zf)/Cerebellum-ZIC1.2-ChIP-Seq(GSE60731)/Homer | 1e-11 | -2.738e+01 | 0.0000 | 2399.0 | 12.63% | 3359.6 | 11.01% | motif file (matrix) | svg |
| 99 | T A G C G C A T A G T C G A T C A T G C G A C T C T A G A C T G A C T G C T G A A C T G C T A G A G T C T G A C C G A T | GLIS3(Zf)/Thyroid-Glis3.GFP-ChIP-Seq(GSE103297)/Homer | 1e-11 | -2.707e+01 | 0.0000 | 2911.0 | 15.32% | 4140.1 | 13.56% | motif file (matrix) | svg |
| 100 | A T G C T C G A T A C G A C G T A T G C A G T C A C G T A G T C A G T C G A T C | Znf263(Zf)/K562-Znf263-ChIP-Seq(GSE31477)/Homer | 1e-11 | -2.678e+01 | 0.0000 | 4396.0 | 23.14% | 6431.1 | 21.07% | motif file (matrix) | svg |
| 101 | A T G C A G T C G T A C A G C T T C G A C T A G G A T C C T G A G T C A A G T C G C T A T C A G | Rfx5(HTH)/GM12878-Rfx5-ChIP-Seq(GSE31477)/Homer | 1e-11 | -2.665e+01 | 0.0000 | 1303.0 | 6.86% | 1730.0 | 5.67% | motif file (matrix) | svg |
| 102 | G A C T C T A G C T A G A G T C T G C A A C T G A C G T A C G T C T A G T C A G | AMYB(HTH)/Testes-AMYB-ChIP-Seq(GSE44588)/Homer | 1e-11 | -2.580e+01 | 0.0000 | 4611.0 | 24.27% | 6778.5 | 22.21% | motif file (matrix) | svg |
| 103 | G T C A G C A T A C T G G T A C G A C T A C T G G C T A A T C G C A G T G T A C C G T A A G C T | Nrf2(bZIP)/Lymphoblast-Nrf2-ChIP-Seq(GSE37589)/Homer | 1e-11 | -2.569e+01 | 0.0000 | 216.0 | 1.14% | 212.0 | 0.69% | motif file (matrix) | svg |
| 104 | C G T A T A G C T A G C T G C A A C T G C T A G C G T A C G T A T C A G G A C T | EHF(ETS)/LoVo-EHF-ChIP-Seq(GSE49402)/Homer | 1e-10 | -2.532e+01 | 0.0000 | 3877.0 | 20.41% | 5645.2 | 18.49% | motif file (matrix) | svg |
| 105 | C A T G T G A C C G T A A G T C T A C G G C A T A C T G G T C A A T G C A G T C | bHLHE41(bHLH)/proB-Bhlhe41-ChIP-Seq(GSE93764)/Homer | 1e-10 | -2.528e+01 | 0.0000 | 2677.0 | 14.09% | 3802.9 | 12.46% | motif file (matrix) | svg |
| 106 | T G A C G T C A A G T C G C T A C T A G A G T C C T G A C A T G A C T G C T A G T A C G T C A G | Zic2(Zf)/ESC-Zic2-ChIP-Seq(SRP197560)/Homer | 1e-10 | -2.497e+01 | 0.0000 | 1026.0 | 5.40% | 1336.8 | 4.38% | motif file (matrix) | svg |
| 107 | A T G C A G T C C T G A A G T C C G A T A C G T A G T C A G T C A C G T A T C G G A C T A C G T | Etv2(ETS)/ES-ER71-ChIP-Seq(GSE59402)/Homer | 1e-10 | -2.475e+01 | 0.0000 | 2712.0 | 14.28% | 3861.5 | 12.65% | motif file (matrix) | svg |
| 108 | C G T A C G T A C G T A G C A T G C A T T A C G G T A C G A C T A C T G C G T A A T C G A C G T G T A C C G T A A G C T | Bach1(bZIP)/K562-Bach1-ChIP-Seq(GSE31477)/Homer | 1e-10 | -2.469e+01 | 0.0000 | 241.0 | 1.27% | 245.6 | 0.80% | motif file (matrix) | svg |
| 109 | G C T A G C A T G A C T G C A T T C A G G T A C G C T A G C A T C T G A G C T A T A G C G C T A C T G A C G A T C T A G | OCT4-SOX2-TCF-NANOG(POU,Homeobox,HMG)/mES-Oct4-ChIP-Seq(GSE11431)/Homer | 1e-10 | -2.360e+01 | 0.0000 | 767.0 | 4.04% | 971.3 | 3.18% | motif file (matrix) | svg |
| 110 | A T G C G A T C G A C T A G C T C G A T C G A T G T C A C G A T T C G A A T C G T A G C T A G C | TATA-Box(TBP)/Promoter/Homer | 1e-9 | -2.262e+01 | 0.0000 | 4655.0 | 24.50% | 6890.2 | 22.57% | motif file (matrix) | svg |
| 111 | T C G A A C G T A C T G C T G A A G T C T C A G A G C T G T A C C G T A A G C T G A T C T C G A | JunD(bZIP)/K562-JunD-ChIP-Seq/Homer | 1e-9 | -2.240e+01 | 0.0000 | 250.0 | 1.32% | 263.6 | 0.86% | motif file (matrix) | svg |
| 112 | G T C A G C A T G C T A C A G T C T A G G A T C C G T A C T G A C G T A C G A T | Oct2(POU,Homeobox)/Bcell-Oct2-ChIP-Seq(GSE21512)/Homer | 1e-9 | -2.193e+01 | 0.0000 | 1290.0 | 6.79% | 1745.1 | 5.72% | motif file (matrix) | svg |
| 113 | C G T A C G T A C G T A A G T C A G C T C T G A A C T G A C T G A G C T A G T C C G T A C T A G C T A G C T A G T G C A | RORa(NR)/Liver-Rora-ChIP-Seq(GSE101115)/Homer | 1e-9 | -2.163e+01 | 0.0000 | 472.0 | 2.48% | 565.5 | 1.85% | motif file (matrix) | svg |
| 114 | C G T A T G A C T A G C T G C A A C T G A C T G C G T A C G T A T C A G G A C T | ELF3(ETS)/PDAC-ELF3-ChIP-Seq(GSE64557)/Homer | 1e-8 | -2.036e+01 | 0.0000 | 2282.0 | 12.01% | 3253.2 | 10.66% | motif file (matrix) | svg |
| 115 | A G C T C T A G T G A C C G T A A C G T C G A T A G T C A G T C C T G A C A T G | TEAD3(TEA)/HepG2-TEAD3-ChIP-Seq(Encode)/Homer | 1e-8 | -2.020e+01 | 0.0000 | 3708.0 | 19.52% | 5450.6 | 17.86% | motif file (matrix) | svg |
| 116 | T C G A T A G C T G C A A C T G A C T G C G T A C G T A C T A G G A C T T A C G | ETS1(ETS)/Jurkat-ETS1-ChIP-Seq(GSE17954)/Homer | 1e-8 | -1.988e+01 | 0.0000 | 2955.0 | 15.56% | 4290.7 | 14.06% | motif file (matrix) | svg |
| 117 | G A C T A G C T G T A C G A C T C T G A A C T G G T C A C T G A A T G C T A C G G A C T A C G T A G T C G A C T C T G A | HRE(HSF)/Striatum-HSF1-ChIP-Seq(GSE38000)/Homer | 1e-8 | -1.848e+01 | 0.0000 | 713.0 | 3.75% | 924.1 | 3.03% | motif file (matrix) | svg |
| 118 | A G T C G C A T C G T A C G T A G T A C A C G T A C T G G A T C G A T C T C G A | BMYB(HTH)/Hela-BMYB-ChIP-Seq(GSE27030)/Homer | 1e-8 | -1.845e+01 | 0.0000 | 4491.0 | 23.64% | 6696.9 | 21.94% | motif file (matrix) | svg |
| 119 | G T C A A C G T C T A G G T C A C G T A G C A T C G T A C G T A G C A T C A G T A G T C C G T A C A G T C T A G C T G A | OCT:OCT(POU,Homeobox,IR1)/NPC-Brn2-ChIP-Seq(GSE35496)/Homer | 1e-7 | -1.787e+01 | 0.0000 | 92.0 | 0.48% | 79.0 | 0.26% | motif file (matrix) | svg |
| 120 | C T G A A C G T A C G T A C G T A G T C G A C T C G A T C T G A A C T G C G T A C G T A T C G A | STAT5(Stat)/mCD4+-Stat5-ChIP-Seq(GSE12346)/Homer | 1e-7 | -1.766e+01 | 0.0000 | 1193.0 | 6.28% | 1637.0 | 5.36% | motif file (matrix) | svg |
| 121 | G C T A A C G T C T A G G T A C G C T A G A C T C T G A G C A T C A T G G A T C | Pit1(Homeobox)/GCrat-Pit1-ChIP-Seq(GSE58009)/Homer | 1e-7 | -1.677e+01 | 0.0000 | 4087.0 | 21.51% | 6092.0 | 19.96% | motif file (matrix) | svg |
| 122 | T G C A C T G A A T G C G T C A A C T G A C T G C G T A C G T A C T A G A G C T | Ets1-distal(ETS)/CD4+-PolII-ChIP-Seq(Barski\_et\_al.)/Homer | 1e-7 | -1.655e+01 | 0.0000 | 949.0 | 5.00% | 1283.4 | 4.20% | motif file (matrix) | svg |
| 123 | G A C T C G A T T C A G G A T C G A C T A G C T A G C T A G T C G A T C C G T A C T A G C T A G T C G A T C G A C T G A | Bcl6(Zf)/Liver-Bcl6-ChIP-Seq(GSE31578)/Homer | 1e-7 | -1.620e+01 | 0.0000 | 4476.0 | 23.56% | 6709.9 | 21.98% | motif file (matrix) | svg |
| 124 | A G T C C T G A A G T C C G A T C A G T G A T C A T G C A C T G A T C G G A C T | Fli1(ETS)/CD8-FLI-ChIP-Seq(GSE20898)/Homer | 1e-6 | -1.608e+01 | 0.0000 | 2877.0 | 15.14% | 4220.2 | 13.83% | motif file (matrix) | svg |
| 125 | G C T A C G T A A G T C A C G T T C G A T A C G A C T G A G C T A G T C T C G A | RORgt(NR)/EL4-RORgt.Flag-ChIP-Seq(GSE56019)/Homer | 1e-6 | -1.590e+01 | 0.0000 | 396.0 | 2.08% | 487.0 | 1.60% | motif file (matrix) | svg |
| 126 | G C T A C G T A A G T C A C G T T C G A T A C G A C T G A G C T A G T C T C G A | RORgt(NR)/EL4-RORgt.Flag-ChIP-Seq(GSE56019)/Homer | 1e-6 | -1.590e+01 | 0.0000 | 396.0 | 2.08% | 487.0 | 1.60% | motif file (matrix) | svg |
| 127 | T A G C C T A G T C G A G A C T A C T G C T G A A G T C T C A G G C A T T G A C C T G A A G C T | Atf7(bZIP)/3T3L1-Atf7-ChIP-Seq(GSE56872)/Homer | 1e-6 | -1.570e+01 | 0.0000 | 1176.0 | 6.19% | 1628.6 | 5.34% | motif file (matrix) | svg |
| 128 | T A C G T A G C G C T A C G A T C T A G A C G T C A G T C A G T G C T A A G T C G T C A G C A T | FOXK2(Forkhead)/U2OS-FOXK2-ChIP-Seq(E-MTAB-2204)/Homer | 1e-6 | -1.545e+01 | 0.0000 | 2079.0 | 10.94% | 3000.4 | 9.83% | motif file (matrix) | svg |
| 129 | T A C G T C G A G A C T A C T G C T G A A G T C T C A G G A C T T G A C C T G A | Atf1(bZIP)/K562-ATF1-ChIP-Seq(GSE31477)/Homer | 1e-6 | -1.534e+01 | 0.0000 | 1710.0 | 9.00% | 2438.1 | 7.99% | motif file (matrix) | svg |
| 130 | C T A G A T G C A T G C C G A T A C T G G A C T A T G C G C T A T G A C A G C T T A G C G C T A | PBX1(Homeobox)/MCF7-PBX1-ChIP-Seq(GSE28007)/Homer | 1e-6 | -1.507e+01 | 0.0000 | 225.0 | 1.18% | 254.9 | 0.84% | motif file (matrix) | svg |
| 131 | T C G A A G C T A C G T A C G T A G T C A G T C A C G T A T C G G A C T A T C G | EWS:ERG-fusion(ETS)/CADO\_ES1-EWS:ERG-ChIP-Seq(SRA014231)/Homer | 1e-6 | -1.496e+01 | 0.0000 | 2342.0 | 12.33% | 3410.2 | 11.17% | motif file (matrix) | svg |
| 132 | C T A G A C T G T G C A G T C A A T G C C G T A A T C G A T G C A G T C C T A G | ZNF341(Zf)/EBV-ZNF341-ChIP-Seq(GSE113194)/Homer | 1e-6 | -1.481e+01 | 0.0000 | 2169.0 | 11.42% | 3146.1 | 10.31% | motif file (matrix) | svg |
| 133 | C G T A C G A T G A C T G A C T T G A C C T G A A T G C C T G A T A G C A G T C A C G T T C G A C A T G T A C G G A C T A T C G G A C T A C G T C T G A T C G A C G T A | Brachyury(T-box)/Mesoendoderm-Brachyury-ChIP-exo(GSE54963)/Homer | 1e-6 | -1.426e+01 | 0.0000 | 945.0 | 4.97% | 1296.6 | 4.25% | motif file (matrix) | svg |
| 134 | A C G T T A C G G A T C A C T G A C G T C T A G A C T G A C T G G A T C C T A G C A T G C T A G | Egr2(Zf)/Thymocytes-Egr2-ChIP-Seq(GSE34254)/Homer | 1e-6 | -1.421e+01 | 0.0000 | 338.0 | 1.78% | 413.6 | 1.35% | motif file (matrix) | svg |
| 135 | C G A T C T A G C T G A A T G C C T G A T C G A C G T A C T G A T C G A T A G C A G T C C G T A A C T G T C G A A T G C | Hand2(bHLH)/Mesoderm-Hand2-ChIP-Seq(GSE61475)/Homer | 1e-6 | -1.408e+01 | 0.0000 | 1577.0 | 8.30% | 2250.4 | 7.37% | motif file (matrix) | svg |
| 136 | T A C G A T G C G A C T A C T G A G C T A G T C G T C A T G C A A C G T A G T C G C T A T G C A | Pknox1(Homeobox)/ES-Prep1-ChIP-Seq(GSE63282)/Homer | 1e-6 | -1.382e+01 | 0.0000 | 616.0 | 3.24% | 814.3 | 2.67% | motif file (matrix) | svg |
| 137 | G C T A A G T C T A C G T G C A A T C G T C A G G C T A T C G A T C A G A G C T | ELF5(ETS)/T47D-ELF5-ChIP-Seq(GSE30407)/Homer | 1e-5 | -1.336e+01 | 0.0000 | 2148.0 | 11.31% | 3133.8 | 10.27% | motif file (matrix) | svg |
| 138 | T C G A G C A T A C G T C T A G G T A C T C G A G C A T T G A C T C G A A C G T | Chop(bZIP)/MEF-Chop-ChIP-Seq(GSE35681)/Homer | 1e-5 | -1.324e+01 | 0.0000 | 663.0 | 3.49% | 887.1 | 2.91% | motif file (matrix) | svg |
| 139 | C T A G C T A G C G T A C G T A T A C G C G A T C T A G C T G A C T G A C G T A T A C G G A C T | PU.1:IRF8(ETS:IRF)/pDC-Irf8-ChIP-Seq(GSE66899)/Homer | 1e-5 | -1.282e+01 | 0.0000 | 620.0 | 3.26% | 827.6 | 2.71% | motif file (matrix) | svg |
| 140 | C T G A A G T C C G A T A G C T A T G C G T A C A C G T A T C G C A G T G C A T | Elf4(ETS)/BMDM-Elf4-ChIP-Seq(GSE88699)/Homer | 1e-5 | -1.217e+01 | 0.0000 | 2788.0 | 14.68% | 4141.9 | 13.57% | motif file (matrix) | svg |
| 141 | T A G C T A G C G A C T C T A G A G C T A G T C G T C A T G C A A C G T A T G C G C T A T G C A | Pbx3(Homeobox)/GM12878-PBX3-ChIP-Seq(GSE32465)/Homer | 1e-5 | -1.207e+01 | 0.0000 | 684.0 | 3.60% | 927.5 | 3.04% | motif file (matrix) | svg |
| 142 | T A G C T A G C G C A T C A T G A C T G G C T A C G T A A C G T A C T G G A T C | TEAD4(TEA)/Tropoblast-Tead4-ChIP-Seq(GSE37350)/Homer | 1e-5 | -1.193e+01 | 0.0000 | 2566.0 | 13.51% | 3800.5 | 12.45% | motif file (matrix) | svg |
| 143 | T G A C A G T C C T G A T G A C C G T A A C G T A C G T A G T C A G T C C G T A | TEAD1(TEAD)/HepG2-TEAD1-ChIP-Seq(Encode)/Homer | 1e-5 | -1.188e+01 | 0.0000 | 3038.0 | 15.99% | 4535.5 | 14.86% | motif file (matrix) | svg |
| 144 | T C A G T A G C G A C T C A T G C T G A A T C G G C A T G T A C C G T A A C T G T A G C T G C A | MafK(bZIP)/C2C12-MafK-ChIP-Seq(GSE36030)/Homer | 1e-5 | -1.172e+01 | 0.0000 | 771.0 | 4.06% | 1059.7 | 3.47% | motif file (matrix) | svg |
| 145 | T C G A C T G A T A G C T G A C T C A G T C A G C G T A C G T A T C A G A G C T | ETV1(ETS)/GIST48-ETV1-ChIP-Seq(GSE22441)/Homer | 1e-5 | -1.168e+01 | 0.0000 | 3696.0 | 19.46% | 5567.0 | 18.24% | motif file (matrix) | svg |
| 146 | C T G A T A C G G C A T A G C T A G C T A G T C T C G A A C T G C A G T A G C T A G C T G A T C | IRF3(IRF)/BMDM-Irf3-ChIP-Seq(GSE67343)/Homer | 1e-5 | -1.155e+01 | 0.0000 | 1171.0 | 6.16% | 1662.3 | 5.45% | motif file (matrix) | svg |
| 147 | T G A C C T A G T C A G G T C A C G T A T C A G C G A T T C A G T C G A T G C A C T G A T A G C | PU.1-IRF(ETS:IRF)/Bcell-PU.1-ChIP-Seq(GSE21512)/Homer | 1e-4 | -1.148e+01 | 0.0000 | 4219.0 | 22.21% | 6392.1 | 20.94% | motif file (matrix) | svg |
| 148 | C G A T G A C T C G A T T C A G G A C T A C G T C A G T C T G A G A C T G A C T A G C T C G A T A C T G A T C G G T A C G C T A | NF1:FOXA1(CTF,Forkhead)/LNCAP-FOXA1-ChIP-Seq(GSE27824)/Homer | 1e-4 | -1.147e+01 | 0.0000 | 224.0 | 1.18% | 267.1 | 0.87% | motif file (matrix) | svg |
| 149 | G C T A C G A T C T A G G T A C G C T A C G A T C G T A G C T A C G A T C A G T G A T C G C T A | Pit1+1bp(Homeobox)/GCrat-Pit1-ChIP-Seq(GSE58009)/Homer | 1e-4 | -1.145e+01 | 0.0000 | 1304.0 | 6.86% | 1865.2 | 6.11% | motif file (matrix) | svg |
| 150 | T C A G T C A G G C T A C G T A T A C G G A C T T C A G T C G A C T G A C G T A T A C G G A C T | IRF8(IRF)/BMDM-IRF8-ChIP-Seq(GSE77884)/Homer | 1e-4 | -1.132e+01 | 0.0000 | 1002.0 | 5.27% | 1409.7 | 4.62% | motif file (matrix) | svg |
| 151 | A C G T C T A G C G T A A G T C G T A C A C G T A C G T A C G T G T C A G T A C T G A C G A C T | Nur77(NR)/K562-NR4A1-ChIP-Seq(GSE31363)/Homer | 1e-4 | -1.116e+01 | 0.0000 | 570.0 | 3.00% | 766.3 | 2.51% | motif file (matrix) | svg |
| 152 | T G C A G C A T C G A T C G T A C A G T A C T G G T A C C G T A C T G A A G C T G T C A A C T G C T A G G T C A C G A T A C T G G T A C T G C A C G T A A G C T | CEBP:CEBP(bZIP)/MEF-Chop-ChIP-Seq(GSE35681)/Homer | 1e-4 | -1.078e+01 | 0.0001 | 487.0 | 2.56% | 647.1 | 2.12% | motif file (matrix) | svg |
| 153 | G A C T C T A G G A T C C A G T A C T G C T G A A T G C G C A T A T G C C T G A | MafA(bZIP)/Islet-MafA-ChIP-Seq(GSE30298)/Homer | 1e-4 | -1.077e+01 | 0.0001 | 2494.0 | 13.13% | 3707.9 | 12.15% | motif file (matrix) | svg |
| 154 | C T A G A C G T A G T C C G T A A C T G A G T C G C A T A C T G G C A T A G T C G A C T G A T C G C A T A G T C A G C T | ZNF317(Zf)/HEK293-ZNF317.GFP-ChIP-Seq(GSE58341)/Homer | 1e-4 | -1.009e+01 | 0.0001 | 248.0 | 1.31% | 307.2 | 1.01% | motif file (matrix) | svg |
| 155 | C G T A C T G A C T A G C G T A C G T A A G T C C G T A C A G T G C A T G T C A C G A T A C T G A C G T G C A T G A T C | PGR(NR)/EndoStromal-PGR-ChIP-Seq(GSE69539)/Homer | 1e-4 | -9.733e+00 | 0.0002 | 818.0 | 4.31% | 1148.5 | 3.76% | motif file (matrix) | svg |
| 156 | C G T A C T G A C G T A C T A G T C G A C T A G A C T G C G T A C G T A T A C G A G C T A T C G | SpiB(ETS)/OCILY3-SPIB-ChIP-Seq(GSE56857)/Homer | 1e-4 | -9.695e+00 | 0.0002 | 665.0 | 3.50% | 919.7 | 3.01% | motif file (matrix) | svg |
| 157 | C A G T A C T G T C A G T G C A G C T A A T G C T C G A A T C G G T C A T G C A | ZNF189(Zf)/HEK293-ZNF189.GFP-ChIP-Seq(GSE58341)/Homer | 1e-4 | -9.608e+00 | 0.0002 | 2564.0 | 13.50% | 3836.7 | 12.57% | motif file (matrix) | svg |
| 158 | A T G C T C A G T C G A G C A T A C T G C G T A A G T C T C A G G A C T T G A C C G T A A G C T | Atf2(bZIP)/3T3L1-Atf2-ChIP-Seq(GSE56872)/Homer | 1e-3 | -9.083e+00 | 0.0003 | 793.0 | 4.17% | 1118.0 | 3.66% | motif file (matrix) | svg |
| 159 | G C A T C T A G G T A C A G T C C G A T A C T G C T A G C T A G G T A C G C T A | ZNF416(Zf)/HEK293-ZNF416.GFP-ChIP-Seq(GSE58341)/Homer | 1e-3 | -8.846e+00 | 0.0004 | 3784.0 | 19.92% | 5763.1 | 18.88% | motif file (matrix) | svg |
| 160 | T C G A G C A T A C T G C T G A A G T C T C A G G A C T G T A C C G T A A G C T A G T C G A T C | c-Jun-CRE(bZIP)/K562-cJun-ChIP-Seq(GSE31477)/Homer | 1e-3 | -8.564e+00 | 0.0005 | 743.0 | 3.91% | 1047.4 | 3.43% | motif file (matrix) | svg |
| 161 | T C A G A T C G G A C T A C T G G A C T C A G T C T A G C G T A G T A C C G T A C T A G A T C G | Tbx20(T-box)/Heart-Tbx20-ChIP-Seq(GSE29636)/Homer | 1e-3 | -8.526e+00 | 0.0005 | 647.0 | 3.41% | 903.9 | 2.96% | motif file (matrix) | svg |
| 162 | T C G A T C G A T A G C G T A C T C A G T A C G C G T A C G T A T C A G A G C T | GABPA(ETS)/Jurkat-GABPa-ChIP-Seq(GSE17954)/Homer | 1e-3 | -8.476e+00 | 0.0006 | 2332.0 | 12.28% | 3495.1 | 11.45% | motif file (matrix) | svg |
| 163 | C T A G C T A G A G T C T C A G A C T G A C G T A C G T C T G A | MYB(HTH)/ERMYB-Myb-ChIPSeq(GSE22095)/Homer | 1e-3 | -8.211e+00 | 0.0007 | 4976.0 | 26.19% | 7662.0 | 25.10% | motif file (matrix) | svg |
| 164 | T C A G C T G A C G T A C G T A T A C G G C A T C T A G C T G A C G T A C G T A T A C G G A C T | IRF1(IRF)/PBMC-IRF1-ChIP-Seq(GSE43036)/Homer | 1e-3 | -7.508e+00 | 0.0015 | 450.0 | 2.37% | 618.9 | 2.03% | motif file (matrix) | svg |
| 165 | C T G A C T A G A T C G G C A T A C T G G T A C A T G C C G T A A C T G G C T A A G T C C G T A | Tbox:Smad(T-box,MAD)/ESCd5-Smad2\_3-ChIP-Seq(GSE29422)/Homer | 1e-3 | -7.442e+00 | 0.0016 | 572.0 | 3.01% | 801.7 | 2.63% | motif file (matrix) | svg |
| 166 | A T G C A G T C G C T A C G A T C G T A G C A T G C T A C G A T C T A G C A T G T G A C G T C A | CArG(MADS)/PUER-Srf-ChIP-Seq(Sullivan\_et\_al.)/Homer | 1e-3 | -7.423e+00 | 0.0016 | 930.0 | 4.90% | 1344.4 | 4.40% | motif file (matrix) | svg |
| 167 | A T C G T G A C A T G C C T G A T C A G G A C T A G T C C G A T T C A G T C G A C A T G C T A G C T A G C G T A C T A G C T A G C T G A C T A G C T A G A T G C | ZSCAN22(Zf)/HEK293-ZSCAN22.GFP-ChIP-Seq(GSE58341)/Homer | 1e-3 | -7.064e+00 | 0.0023 | 157.0 | 0.83% | 194.7 | 0.64% | motif file (matrix) | svg |
| 168 | T A C G C T G A T C G A C G A T C T A G C T A G T C G A C T G A T C G A T C G A C G T A T C G A G C A T C A T G C G T A T A C G G C A T T G A C C G T A A G C T | NFAT:AP1(RHD,bZIP)/Jurkat-NFATC1-ChIP-Seq(Jolma\_et\_al.)/Homer | 1e-3 | -6.973e+00 | 0.0025 | 515.0 | 2.71% | 720.5 | 2.36% | motif file (matrix) | svg |
| 169 | C T A G T A C G G A C T T G C A T G C A C G A T T A C G C T G A T C G A C T G A | Hoxa10(Homeobox)/ChickenMSG-Hoxa10.Flag-ChIP-Seq(GSE86088)/Homer | 1e-2 | -6.855e+00 | 0.0027 | 1864.0 | 9.81% | 2796.6 | 9.16% | motif file (matrix) | svg |
| 170 | T A G C G A T C G T A C C T G A G C A T C T G A C G T A T G C A C G T A G A T C | Hoxa13(Homeobox)/ChickenMSG-Hoxa13.Flag-ChIP-Seq(GSE86088)/Homer | 1e-2 | -6.813e+00 | 0.0028 | 8561.0 | 45.07% | 13418.3 | 43.96% | motif file (matrix) | svg |
| 171 | C A T G A C G T A G T C G A T C G A T C G A T C G C T A C T A G C T A G C T A G T C A G T C G A | EBF1(EBF)/Near-E2A-ChIP-Seq(GSE21512)/Homer | 1e-2 | -6.807e+00 | 0.0028 | 2139.0 | 11.26% | 3226.9 | 10.57% | motif file (matrix) | svg |
| 172 | T G A C C T G A C T A G C T G A C G T A A G T C C T G A A C G T G C A T T A G C G C A T A T C G G A C T G A C T G A T C | GRE(NR),IR3/RAW264.7-GRE-ChIP-Seq(Unpublished)/Homer | 1e-2 | -6.718e+00 | 0.0031 | 715.0 | 3.76% | 1025.4 | 3.36% | motif file (matrix) | svg |
| 173 | A T G C G A C T A G C T A G C T A G T C G C T A C A G T C G A T G C T A A C G T A C T G G C T A T A G C G C A T T G A C | IRF:BATF(IRF:bZIP)/pDC-Irf8-ChIP-Seq(GSE66899)/Homer | 1e-2 | -6.544e+00 | 0.0037 | 396.0 | 2.08% | 547.0 | 1.79% | motif file (matrix) | svg |
| 174 | C T G A A T G C C G T A A C G T A G T C A G T C A C G T A C T G A T C G G C A T | SPDEF(ETS)/VCaP-SPDEF-ChIP-Seq(SRA014231)/Homer | 1e-2 | -6.243e+00 | 0.0049 | 2931.0 | 15.43% | 4481.2 | 14.68% | motif file (matrix) | svg |
| 175 | C T G A T A G C T G A C T C A G C T A G G T C A C G T A T C A G A G C T T C A G | ETV4(ETS)/HepG2-ETV4-ChIP-Seq(ENCODE)/Homer | 1e-2 | -5.835e+00 | 0.0074 | 2659.0 | 14.00% | 4063.2 | 13.31% | motif file (matrix) | svg |
| 176 | C G T A C G T A C G T A G C A T G C A T A C T G G T A C G A C T C T A G G C T A T A C G G A C T T G A C C G T A A G C T | NFE2L2(bZIP)/HepG2-NFE2L2-ChIP-Seq(Encode)/Homer | 1e-2 | -5.799e+00 | 0.0076 | 261.0 | 1.37% | 352.2 | 1.15% | motif file (matrix) | svg |
| 177 | A T G C C T G A A T C G T A C G A G T C C G A T T C A G C G A T C T A G A G C T G T C A G T C A C G T A A G T C C G T A T A C G C T G A | Fox:Ebox(Forkhead,bHLH)/Panc1-Foxa2-ChIP-Seq(GSE47459)/Homer | 1e-2 | -5.676e+00 | 0.0085 | 3544.0 | 18.66% | 5463.6 | 17.90% | motif file (matrix) | svg |
| 178 | C G T A C A T G C A T G A C T G C T A G T C G A G C A T C G A T A G C T A G T C G A T C G T A C | NFkB-p65(RHD)/GM12787-p65-ChIP-Seq(GSE19485)/Homer | 1e-2 | -5.541e+00 | 0.0097 | 1177.0 | 6.20% | 1752.2 | 5.74% | motif file (matrix) | svg |
| 179 | G A T C G T A C C G A T A C T G A C T G C G T A C G T A A C G T A C T G G A T C | TEAD(TEA)/Fibroblast-PU.1-ChIP-Seq(Unpublished)/Homer | 1e-2 | -5.459e+00 | 0.0105 | 2146.0 | 11.30% | 3266.5 | 10.70% | motif file (matrix) | svg |
| 180 | A T G C T A G C A G C T A G C T T G A C G A C T T C A G T A C G G T C A C T G A A T C G T A G C G A C T C A G T A G T C A G C T T C G A A T C G T G C A T G C A | HRE(HSF)/HepG2-HSF1-ChIP-Seq(GSE31477)/Homer | 1e-2 | -5.313e+00 | 0.0120 | 395.0 | 2.08% | 556.1 | 1.82% | motif file (matrix) | svg |
| 181 | C T G A A T C G A G C T A G C T A C G T T A G C C T G A T A C G C G A T A C G T G A C T A G T C | ISRE(IRF)/ThioMac-LPS-Expression(GSE23622)/Homer | 1e-2 | -4.905e+00 | 0.0180 | 213.0 | 1.12% | 288.0 | 0.94% | motif file (matrix) | svg |
| 182 | C A T G G A C T G C A T A C T G A G C T A C T G A C T G C G T A G C A T A G C T A T C G T A C G | Foxh1(Forkhead)/hESC-FOXH1-ChIP-Seq(GSE29422)/Homer | 1e-2 | -4.847e+00 | 0.0190 | 1859.0 | 9.79% | 2830.2 | 9.27% | motif file (matrix) | svg |
| 183 | G A T C T C G A A G T C C G A T C G A T A G T C A T G C A C T G A T C G G A C T | Elk1(ETS)/Hela-Elk1-ChIP-Seq(GSE31477)/Homer | 1e-2 | -4.789e+00 | 0.0200 | 1095.0 | 5.76% | 1638.1 | 5.37% | motif file (matrix) | svg |
| 184 | T G C A C G T A A C T G T C A G C A G T C A T G T C A G G A T C T A C G A G T C T G C A A C T G A C T G T G A C G T C A | ZNF165(Zf)/WHIM12-ZNF165-ChIP-Seq(GSE65937)/Homer | 1e-2 | -4.786e+00 | 0.0200 | 209.0 | 1.10% | 283.6 | 0.93% | motif file (matrix) | svg |
| 185 | C G T A T A C G T C G A A C T G A C T G C G T A C G T A T A C G A G C T T A C G | PU.1(ETS)/ThioMac-PU.1-ChIP-Seq(GSE21512)/Homer | 1e-2 | -4.756e+00 | 0.0205 | 1397.0 | 7.35% | 2109.1 | 6.91% | motif file (matrix) | svg |
| 186 | C T A G A T C G G A C T A G T C A G T C A G T C G A C T C T G A A C T G C T A G A C T G C T G A | EBF(EBF)/proBcell-EBF-ChIP-Seq(GSE21978)/Homer | 1e-2 | -4.658e+00 | 0.0224 | 459.0 | 2.42% | 660.9 | 2.17% | motif file (matrix) | svg |
